# IoTDI-ImbS: A Precise Identification Model and Algorithm for IoT Devices from Network Traffic

**DOI:** 10.3390/s26113530

**Published:** 2026-06-03

**Authors:** Junhao Qian, Shuang Zhao, Zhihao Wang, Zhihua Li

**Affiliations:** 1 School of Automation and Intelligent Science, Jiangnan University, Wuxi 214122, China; qjhao@jiangnan.edu.cn; 2 School of Artificial Intelligence and Computer Science, Jiangnan University, Wuxi 214122, China; zhaoshuang262026@163.com (S.Z.); zhihaowang2@163.com (Z.W.)

**Keywords:** network traffic, bidirectional long short-term memory neural network (BiLSTM), residual network 18 (ResNet18), IoT device identification

## Abstract

With the rapid development of the Internet of Things (IoT) and the increase in the frequency of cyberattacks, accurate identification of IoT end devices is critical to their security. Existing identification methods are based on raw, statistical, and deep features of network traffic, each with their own advantages and disadvantages. Raw feature-based methods have difficulty performing feature extraction and insufficient information. As such, the recognition accuracy of statistical feature-based methods is limited by the distinguishment machine learning classifiers, and the deep feature-based methods do not take into account the problem of large differences in traffic samples, which leads to low recognition accuracy in some devices. For this reason, this paper proposes the IoTDI-ImbS method. The method selects the network traffic payload information as the original features and converts them into grayscale images; uses a generative adversarial network-based IoT terminal devices traffic generation (NTGAN) algorithm to generate traffic samples for devices with fewer samples through generative adversarial network to solve the sample imbalance problem; and constructs a ResNet18-BiLSTM model, mining spatial features with ResNet18 and extracting temporal features with BiLSTM to improve recognition accuracy. The experimental results on different sizes of IoT terminal device datasets show that IoTDI-ImbS has performance advantages over other methods in recognition accuracy, better leverages the sample imbalance problem in the dataset, and provides a more effective solution for IoT device recognition. Experimental results on the UNSW and IoT Sentinel dataset demonstrate that IoTDI-ImbS significantly outperforms baseline methods. Specifically, on the UNSW dataset, our method achieves an overall accuracy of 99.1% and an F1-score of 0.985. After integrating the NTGAN module, the identification accuracy for minority classes improved by approximately 3.5%. On the IoT Sentinel dataset, the model maintains a high precision of 98.7%, proving its robustness in diverse IoT environments.

## 1. Introduction

According to information released by the Data Institute, more than 7.5 billion terminal devices worldwide are expected to be connected to the Internet of Things (IoT) by 2025, and the global expenditure of the IoT will exceed 1.1 trillion USD. In addition, the amount of data transfer between IoT terminal devices will reach a staggering 79.4 ZB [1]. At present, protection of data privacy and security in IoT terminal devices is a major challenge. In an IoT environment, protecting all IoT terminal devices would significantly increase the security overhead. A reasonable solution is to protect only a few specific target devices. To reduce the security protection overhead, these target devices need to be accurately identified. Identifying IoT terminal devices from their network traffic is an important research method that is crucial for the security of target devices [2]. The aforementioned measure can efficiently reduce the security protection overhead while protecting the security and privacy of the target devices. This will provide a more reliable foundation for the development of the IoT and promote the widespread application of IoT technologies.

Previous studies [3,4,5,6,7,8,9,10,11,12,13,14,15,16,17,18,19,20,21,22,23,24] have shown that IoT terminal device identification methods and models can be classified based on (i) raw features, (ii) statistical features, and (iii) deep features of the network traffic. In [3,4,5,6,7,8,9], IoT terminal devices were identified based on the raw features of the network traffic, such as the size, direction, and protocol header information of network traffic packets. However, extracting these features is difficult, and the information available is insufficient; thus, the accuracy of identifying IoT terminal devices using this method must be improved. In [10,11,12,13,14,15,16,17], the features in the network traffic were counted and analyzed, and these data were used to reflect the traffic patterns and behavioral features of different IoT end devices; however, the recognition accuracy of this method was poor because machine learning classifiers were employed to identify IoT terminal devices. In [18,19,20,21,22,23,24], IoT terminal devices were identified based on the deep features of the network traffic. A deep learning model was used to extract the deep features from the network traffic to efficiently capture the differences and behavioral patterns between devices, and experimental results showed that the recognition accuracy of the IoT terminal device identification method was significantly improved.

However, in previous studies [3,4,5,6,7,8,9], the extracted raw traffic feature information on IoT terminal devices was insufficient, leading to the poor recognition accuracy of these devices. By contrast, in  [10,11,12,13,14,15,16,17,18], the raw traffic feature information on IoT terminal devices was added by statistically analyzing the feature information from the traffic generated by these devices. However, only machine learning algorithms were employed for classification; this resulted in the low recognition accuracy of the IoT terminal devices. In [19,20,21,22,23,24,25], deep learning models were used to learn the implicit feature information in the traffic of the IoT terminal devices to improve recognition accuracy. However, the fact that traffic samples generated by IoT terminal devices vary widely was not considered, leading to the insufficient recognition accuracy of some IoT terminal devices. To overcome the aforementioned limitations, in this study, the payload information in the network traffic is selected as the raw traffic feature of the IoT terminal devices. Subsequently, the network traffic information is preprocessed, and the data are transformed into a grayscale image having a uniform format as the input of the neural network model. A generative adversarial network (GAN) is used to generate network traffic samples for IoT terminal devices with few samples. Finally, a deep residual network (ResNet18) and bidirectional long short-term memory (BiLSTM) are integrated, and a ResNet18-BiLSTM-based IoT terminal device identification (ResNet18-BiLSTM-based DI) model is proposed to improve the accuracy of the IoT terminal device identification methods.

In summary, this paper presents an IoT terminal device identification method for imbalanced samples (IoTDI-ImbS). The payload information in the IoT terminal device traffic is selected as the raw traffic feature. The payload information is then transformed into a grayscale image through data preprocessing. A generative adversarial network-based IoT terminal device traffic generation (NTGAN) algorithm is proposed to balance traffic samples for IoT terminal devices. Finally, a ResNet18-BiLSTM-based DI model is proposed for IoT terminal device identification. The main contributions of this study are as follows:

- We propose a Payload-to-Image conversion mechanism that transforms non-structural traffic into grayscale images, enabling the extraction of high-dimensional spatial patterns that raw byte sequences cannot easily represent.
- We develop the NTGAN algorithm with a loss threshold filtering mechanism. It specifically targets the data scarcity of minority IoT classes, effectively mitigating model bias caused by severe class imbalance.
- We design a ResNet18-BiLSTM spatiotemporal model. By fusing CNN-based spatial features with RNN-based temporal dependencies, it achieves superior identification precision compared to single-stream deep learning models.

For this reason, this paper proposes the IoTDI-ImbS method, designed as a synergistic three-stage pipeline to overcome these challenges. First, we implement a Payload-to-Image conversion to transform unstructured byte streams into spatial grayscale representations, enabling the model to leverage advanced computer vision features. Second, to tackle the inherent data imbalance among IoT devices, the NTGAN algorithm is integrated to perform targeted data augmentation within this image domain. Finally, the ResNet18-BiLSTM architecture is employed as a unified classifier, where ResNet extracts local spatial textures and BiLSTM captures the sequential temporal dependencies of the traffic. This integrated approach ensures both data balance and comprehensive feature learning.

## 2. Related Works

In [3,4,5,6,7,8,9,10,11,12,13,14,15,16,17,18,19], three types of IoT terminal device identification methods were proposed: methods based on the raw, statistical, and deep features of the network traffic.

### 2.1. IoT Terminal Device Identification Methods Based on the Raw Features of the Network Traffic

IoT terminal device identification methods based on the raw features of the network traffic extract key features of device communication, such as the packet length, communication frequency, and protocol information. Machine learning is used to accurately classify different IoT terminal devices. The advantage of these methods is that the information is obtained directly from the communication behavior, which helps to monitor the network traffic in real time and improves the adaptability of the system to new devices.

Ref. [3] proposed an entropy-based IoT terminal device identification method. They calculated the traffic entropy of each IoT terminal device and used a machine learning algorithm to identify the IoT terminal device; the IoT identification accuracy rate reached 94%. Ref. [4] used the network traffic of data packets having a specific length sent by IoT terminal devices for identification. Although this method improved the identification efficiency, its generalization ability was limited. Ref. [5] used the domain name system traffic information from IoT terminal devices as the raw traffic features and designed a multi-label classifier named IoTFinder for identification.

Ref. [6] proposed a traffic-based Nilsimsa hash to recognize IoT terminal devices with 93% accuracy rate and 90% recall rate. Ref. [7] proposed a sliding window-based IoT terminal device identification method, in which the network traffic was divided into multiple sub-flows through a sliding window. The original traffic features in the network traffic were extracted as the raw traffic features of these devices. Finally, the random forest algorithm was used to complete the identification of the IoT terminal devices, with an accuracy of 96.23% and a recall rate of 91.47%.

### 2.2. IoT Terminal Device Identification Methods Based on the Statistical Features of the Network Traffic

IoT terminal device identification methods based on the raw features of the network traffic exhibit good identification capability. However, their identification efficiency is low and scope of application is limited. By contrast, IoT terminal device identification methods based on the statistical features of the network traffic can accurately classify the different devices according to the type by analyzing the statistical information on device communication, such as the data transfer rate, connection duration, and communication frequency. With the help of machine learning or deep learning models, the methods can learn the communication behavior of the device from these statistical features and improve the recognition accuracy. These methods work in real time, are efficient, and can quickly adapt to changes in the device behavior.

Ref. [10] proposed a machine learning-based IoT terminal device identification method, IoTDevID. First, the network traffic data packets were statistically analyzed, and the feature information was extracted and used as the raw traffic feature of the IoT terminal devices. This information was input into a machine learning model to classify the IoT terminal devices. Ref. [11] proposed a device type identification method based on traffic classification. The statistical features of the first part of a message in the data packet and the payload features were used as the raw traffic features. The random forest algorithm was further used to complete the device identification. This method classified only some of the instrumentation and control IoT terminal devices. Ref. [12] used 23-dimensional network traffic features from the network traffic data packets as raw IoT terminal device features and used machine learning algorithms to classify 27 types of IoT terminal devices; however, the accuracy of 10 of the IoT terminal devices was only approximately 50%.

### 2.3. IoT Terminal Device Identification Methods Based on the Deep Features of the Network Traffic

Although the accuracy of IoT terminal device identification methods based on the statistical features of the network traffic has considerably improved, these methods lack adaptability. In contrast, IoT terminal device identification methods based on the deep features of the network traffic utilize deep learning techniques to comprehensively assess the details of the communication data obtained from devices, such as protocol headers and packet contents, to extract high-level information. IoT terminal devices are accurately classified and identified by learning the communication behavior, patterns, and identifiers of the devices using deep learning models. This approach not only adapts to encrypted traffic and complex communication patterns but also provides a stronger protection mechanism for network security and improves the accuracy and robustness toward device identification. Ref. [19] proposed an IoT terminal device recognition method named CBBI, which was used to extract the temporal and spatial features in the network traffic. A data enhancement module for balancing the dataset samples was proposed to improve the recognition accuracy of IoT terminal devices. However, the federated GAN in the data enhancement module was not improved, which resulted in poor accuracy in the recognition of some of the IoT terminal devices. Ref. [20] proposed an end-to-end IoT terminal device recognition method using convolutional neural network and BiLSTM neural network models to extract features from the network traffic, ultimately achieving the recognition of IoT terminal devices. However, their experimental results indicated that certain IoT terminal devices were poorly recognized. Ref. [21] proposed an IoT terminal device recognition model based on depthwise separable convolution, which replaced the standard convolution to improve the recognition accuracy of IoT terminal devices. However, this work also failed to solve the problem of imbalanced traffic samples in the IoT terminal device dataset, which resulted in the low recognition accuracy for some of the IoT terminal devices.

### 2.4. Differences Between the Research in This Article and Existing Studies

This study addresses two critical challenges in IoT terminal device recognition: sample imbalance and low recognition accuracy. Compared with the existing work, it introduces innovations in three key areas: feature selection, sample balancing, and model construction.

First, in feature selection, prior methods using primitive traffic features suffer from extraction difficulties and limited information, while statistical approaches are also affected by imbalance. In contrast, we use network payload data—which better reflects device behavior—as raw features, converting them into grayscale images for model input.

Second, to address class imbalance, we propose NTGAN, a generative adversarial network designed to augment samples for under-represented devices. NTGAN introduces a loss-value threshold to filter low-quality synthetic data, thereby improving sample diversity and distribution.

Third, prior deep learning methods often neglect both imbalance and spatio-temporal features in the model architecture. Our ResNet18-BiLSTM model captures spatial and sequential traffic characteristics and incorporates an attention mechanism to enhance focus on critical patterns, improving recognition performance especially for low-frequency devices.

## 3. IoTDI-ImbS

**Figure 1:**
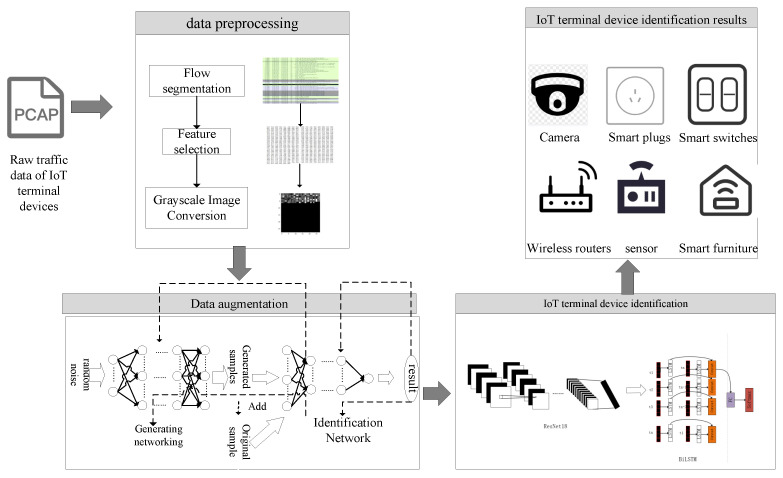
The logical framework for identifying IoT terminal devices.

The logical framework of the IoTDI-ImbS is shown in Figure 1. It includes three main phases: data preprocessing, data enhancement, and IoT terminal device identification. In the data preprocessing stage, the raw traffic data from IoT terminal device is analyzed and the payload information from the network traffic is used as the raw traffic feature of the IoT terminal device. Subsequently, the payload data, selected based on their features, is converted into grayscale images that are used as inputs to the neural network model. The data enhancement phase focuses on addressing the sample imbalance that exists in the raw IoT terminal device traffic dataset. The NTGAN algorithm is applied to generate additional traffic samples for IoT terminal devices with few samples in the raw traffic dataset, ensuring that the model can comprehensively learn the features of all sample types during the training process. In the IoT terminal device identification phase, the spatial features of the raw IoT terminal device traffic data are first extracted by using the ResNet18, and then, the temporal features are extracted by using the BiLSTM network. Finally, the IoT terminal devices are accurately identified by employing the ResNet18-BiLSTM-based DI model. This integrated model efficiently combines the spatial and temporal features to improve the identification accuracy of the IoT terminal devices.

The proposed IoTDI-ImbS framework consists of three interconnected modules: data preprocessing (conversion to images), data balancing (NTGAN), and device identification (ResNet18-BiLSTM). As illustrated in Figure 1, these components do not function in isolation; the grayscale images generated in the first stage provide the necessary visual format for NTGAN to synthesize minority class samples, which collectively form a balanced and high-dimensional input for the subsequent spatiotemporal feature extraction in the ResNet18-BiLSTM model.

### 3.1. Data Preprocessing

**Figure 2:**
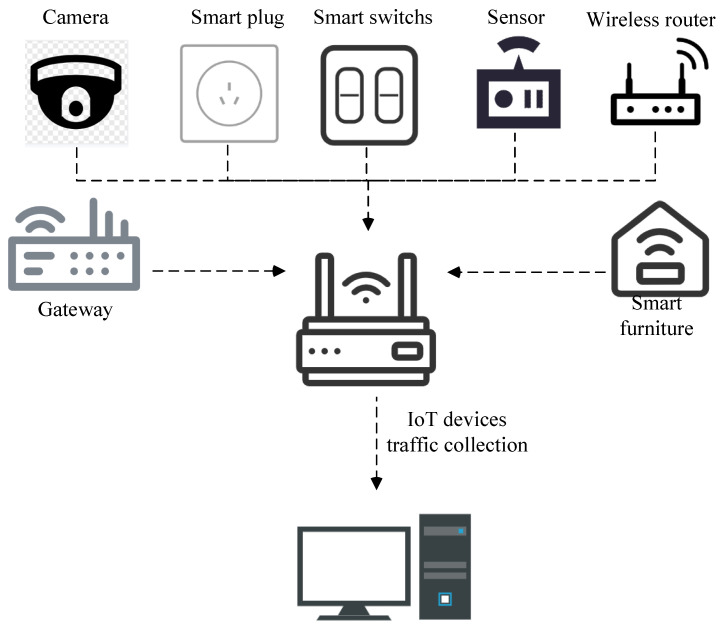
IoT terminal device traffic collection diagram.

The collection of IoT terminal device traffic aims to identify device behavior by analyzing communication data, enhancing system security and performance management, and is an essential means to ensure the normal operation and data security of IoT terminal devices. To this end, traffic information from IoT terminal devices in the IoT environment was collected by using tools such as Wireshark, as illustrated in Figure 2, which depicts the IoT terminal device traffic collection process. In this study, the IoT terminal device traffic datasets employed in [12,26] were primarily used to accomplish the goal of IoT terminal device identification. The raw IoT terminal device traffic in the dataset was stored in the pcap file format and had the following features:

- The raw IoT terminal device traffic file contained multiple traffic packets from these devices. Therefore, the file size was large, which increased the processing time required for data preprocessing.
- Given the large amount of redundant information in the IoT terminal device traffic packets, directly using the raw traffic data as the raw features would reduce the recognition efficiency of the IoT terminal device identification model.
- The data in the IoT terminal device traffic file was stored in the hexadecimal format, which cannot be directly used as input for neural network models. Synthetic image generation is more mature and stable than generating long, discrete 1D sequences, ensuring higher quality samples for balancing the dataset.
- By leveraging CNN architectures (like ResNet18) on image data, the model gains translation invariance. This means it can recognize specific device fingerprints even if their position shifts slightly within the payload.

**Figure 3:**
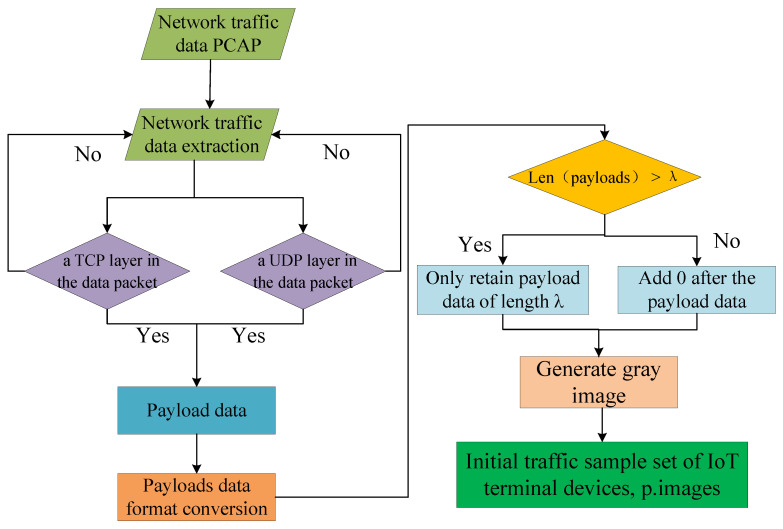
Flowchart of the data preprocessing algorithm.

Therefore, a data preprocessing algorithm based on the features of IoT terminal device traffic files was proposed, and its work flow is shown in Figure 3. The data-preprocessing method consisted of three main parts: traffic segmentation, feature selection, and grayscale image generation. The pseudo-code of data preprocessing is shown in Algorithm 1. It defines the original IoT terminal device traffic as *T*; the file *T* is in pcap format. The sample set of IoT terminal device traffic files was obtained after data preprocessing. In the Payload-to-Image conversion stage, raw packets are normalized and mapped into grayscale pixel values. This process functions as a form of lossy abstraction, making it computationally difficult to reconstruct the original sensitive plaintext from the generated images.

**Algorithm 1:** DPP Algorithm
    **Input:** *T*//Original IoT terminal device network traffic file    **Output:** Files
  **1**SplitCap(*T*)→$t_{1}t_{2}t_{3}\cdots t_{i}$  **2****for** *$t_{i}$ in t* **do**  **3**       lists = rdp($t_{i}$)  **4**       **for** *each list in lists* **do**  **5**              **if** *list.haslayer(TCP)* **then**  **6**                      Payload = list[TCP].payload.original  **7**              
**end**
  **8**              **if** *list.haslayer(UDP)* **then**  **9**                      Payload = list[UDP].payload.original**10**              
**end**
**11**              temp = HexToDec(Payload) Payload_list.append(temp)**12**       
**end**
**13**       **for** *each plist in Payload_list* **do****14**              **if** *len(plist) >= $\lambda_{len}$* **then****15**                      File_list.append(plist[0: $\lambda_{len}$])**16**              
**end**
**17**              **if** *len(plist) < $\lambda_{len}$* **then****18**                      File_list.append(pad(plist, $\lambda_{len}$))**19**              
**end**
**20**       
**end**
**21**       **for** *each file in File_list* **do****22**              $File\text{\_}list_{i}$→$png_{i}$ $\mathrm{pn}g_{1}\cup \mathrm{pn}g_{2}\cup \mathrm{pn}g_{3}\cdots \cup \mathrm{pn}g_{i}$→$Files_{t_{i}}$**23**       
**end**
**24** 
**end**
**25** **return** Files


#### 3.1.1. Flow Segmentation

To extract the traffic files of different devices from the hybrid IoT terminal device traffic files, they should be segmented based on the quintuple information in the IoT terminal device traffic data packets. The SplitCap tool was utilized in this study to achieve this goal. Traffic segmentation enables IoT terminal device identification models to quickly and accurately process the traffic data from different IoT terminal devices.

#### 3.1.2. Feature Selection

An in-depth analysis of the raw IoT terminal device traffic was conducted to improve the accuracy and efficiency of the IoT terminal device identification model. The payload information in the traffic packets from IoT terminal devices was fully utilized because the raw traffic data features plays an important role in improving the recognition performance of the method. The reasons for this are as follows:

- IoT terminal devices usually use specific protocols and application layer data formats when communicating with each other. In this process, the payload information contains identifiable patterns and data structures related to different IoT terminal devices. Analyzing this information in depth helps in efficiently distinguishing the communication features between different IoT terminal devices.
- Different IoT terminal devices may apply communication protocols in unique ways, resulting in differences in the payload information in the IoT terminal device traffic. Adequate consideration of these differences allows for more accurate identification of IoT terminal device types and IoT terminal device manufacturer information, which can be used to improve the accuracy of the overall IoT terminal device identification model.
- The payload information from the IoT terminal device traffic data packets can be used to detect potential security threats to the IoT terminal device or any abnormal behavior of the device. By monitoring this information, the potential security risks can be identified in a timely manner, thus enhancing the security of the IoT system.

Overall, the payload information from the raw IoT terminal device traffic was selected as the raw traffic feature device identification to shorten the identification time.

#### 3.1.3. Grayscale Image Conversion

The payload data extracted from the feature selection of the IoT terminal device traffic are not directly available as input to the deep learning models. This is because payload data are stored in the hexadecimal format in the IoT terminal device traffic and need to be converted to decimal format first.

**Figure 4:**
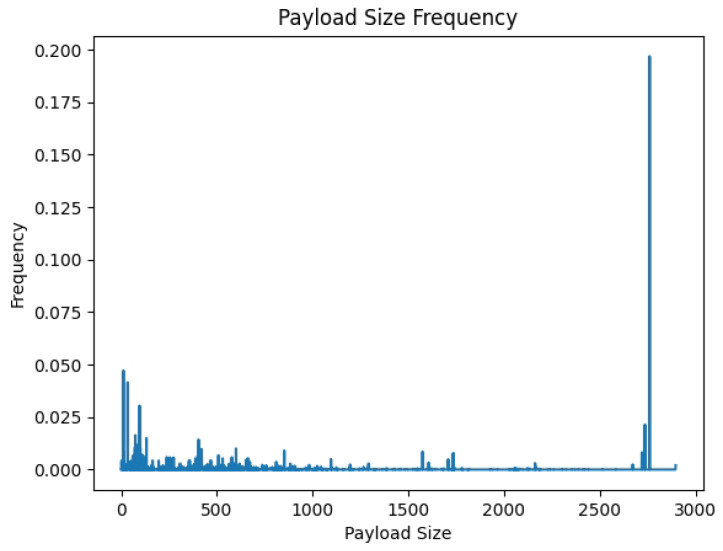
The IoT Sentinel dataset payload length distribution information.

**Figure 5:**
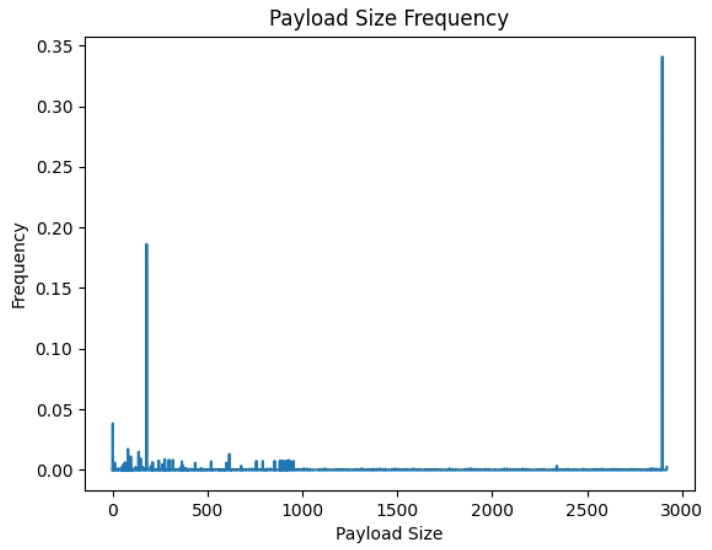
The UNSW dataset payload length distribution information.

**Figure 6:**
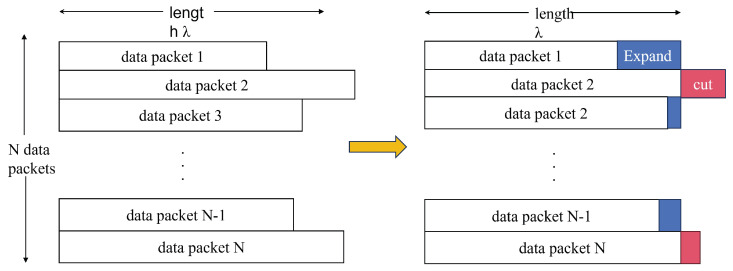
The data packet payload data length adjustment diagram.

Furthermore, as depicted in Figure 3, payload data lengths vary in the decimal format in the network traffic packets. To ensure the input consistency of the IoT terminal device identification model, the following steps were implemented: first, the payload information data were converted into the decimal format. Next, the length thresholds of the payloads were set to λ depending on the payload data length in the different IoT terminal device traffic datasets displayed in Figure 4 and Figure 5. The payload lengths of the different data packets in the IoT terminal device traffic were processed as follows (Figure 6):

- When the payload data length of the packet in the IoT terminal device traffic was greater than λ, the payload information was clipped.
- When the payload data length of the packet in the IoT terminal device traffic was less than λ, a zero-fill operation was performed on the payload information. Finally, the processed payload data in the terminal device traffic with unified lengths were converted into grayscale images, where the pixel values were mapped one by one to complete the grayscale image transformation. This process ensured consistency of the input data length of the IoT terminal device identification model, providing a uniform input for accurate identification of the IoT terminal device model at the specific device model level.

### 3.2. Data Augmentation

With the completion of data preprocessing, IoT terminal device traffic in the dataset was successfully converted from pcap file format to traffic data samples with uniformly formatted grayscale image format. However, different IoT terminal devices come from different manufacturers and have unique functions, hardware and software compositions, which led to huge differences in the size of the network traffic they generate. For example, network traffic generated by surveillance devices (e.g., cameras) contained a large number of network traffic data packets, whereas traffic generated by other sensory devices contained few packets. In addition, in the smart home domain, the network traffic generated by devices such as smart light bulbs and smart sockets contained a smaller number of network traffic data packets. As a result, there were large differences in the number of IoT terminal device traffic data samples produced from different IoT terminal device traffic on performing data preprocessing.

When traffic data samples from IoT terminal devices are fed into a neural network model for training, it is often assumed that the model treats these samples as coming from a real distribution pattern and considers them unbiased. Therefore, a significant difference in the number of IoT terminal device traffic samples in the training set might have an impact on the results of the IoT terminal device recognition model. Specifically, IoT terminal devices that feature a large number of traffic samples are more likely to be correctly identified by the model, whereas those with relatively few traffic samples are more prone to being misidentified as devices with a higher volume of traffic samples. Therefore, to address the problem of unbalanced traffic samples for IoT terminal devices, the dataset generated via data preprocessing has been adjusted to improve the identification accuracy.

GAN was used to generate additional traffic samples for IoT terminal devices with a relatively small sample size in the dataset. GAN is a deep learning framework proposed by Ian Goodfellow in 2014. It is unique because it is based on two mutually adversarial neural network models: the generator and the discriminator. The network is based on the concept of “adversarial games” in game theory; during the training process, the generator and the discriminator compete against each other similar to two players. In this process, the goal of the generator is to produce synthetic samples that are similar to real traffic samples, whereas the task of the discriminator is distinguishing between real and synthetic samples as accurately as possible. This competitive dynamic enables the generator to gradually learn to generate more realistic traffic samples to confuse the discriminator, whereas the discriminator continuously improves its ability to effectively distinguish between genuine and fake samples.

We employed the NTGAN-based augmentation rather than simpler methods like random over-sampling (ROS) for several reasons:

- Preventing Overfitting: Simple over-sampling replicates existing minority samples, which often leads to overfitting as the model merely memorizes specific data points. In contrast, NTGAN generates new, synthetic samples that follow the underlying distribution of the minority class, enhancing the model’s generalization.
- Preserving Feature Diversity: Unlike SMOTE or ROS, which may introduce noise or blur in the image domain, NTGAN captures the high-dimensional latent patterns of IoT traffic images. It ensures that the generated ’synthetic traffic’ maintains the structural integrity and statistical characteristics required for precise fingerprinting.
- Improved Stability: By incorporating a loss-threshold filtering mechanism, NTGAN filters out low-quality or non-representative samples, ensuring that the augmented dataset is both balanced and high-fidelity.

GAN was used in this study to generate additional traffic samples for balancing the distribution of traffic samples of IoT terminal devices in the dataset, thereby improving the performance of identifying devices with a low number of samples. The input to the generator was arbitrary noise, $P_{z}\left(z\right)$, and a batch of spurious data, $G\left(z\right)$, was generated. The loss function during generator training was $L_{G}$. The inputs to the discriminator were spurious data samples generated by the generator and real data samples, and the task of the discriminator was to distinguish whether the data was from the real sample dataset or the spurious sample set from the generator. Assume that the discriminator input parameter is *x*, *x* is from $P_{data}\left(x\right)$, the output of the discriminator, $D\left(x\right)$, is the probability of belonging to a real data sample, and the loss function generated during the training of the discriminator is $L_{D}$. The goals of the generator and discriminator are to generate data samples that are as realistic as possible through continuous adversarial actions, i.e., the generator generates spurious data samples that are as realistic as possible to deceive the discriminator, whereas the discriminator learns to more accurately recognize the deceptive data in *x*. The objective function *V*, of the GAN is expressed in Equation (1):

(1) $$\begin{array}{cc} \min_{G}\max_{D}V\left(D,G\right) & =E_{x∼P_{\mathrm{data}}\left(x\right)}\left[\log D(x)\right]\\ \; & +E_{z∼P_{z}\left(z\right)}\left[\log\left(1-D(G(z))\right)\right] \end{array}$$

When NTGAN is trained, the loss values, $L_{G}$ and $L_{D}$, of the generator and discriminator, respectively, are calculated by applying Equations (2) and (3):

(2) $$L_{G}=-\frac{1}{m}\sum_{i=1}^{m}\log\left(D\left(G\left(z^{i}\right)\right)\right)$$

(3) $$L_{D}=-\frac{1}{m}\sum_{i=1}^{m}\left[\log\left(D\left(x^{i}\right)\right)+\log\left(1-D\left(G\left(z^{i}\right)\right)\right)\right]$$

where *m* is the number of samples in each batch during the training process, $z^{i}$ is the noise vector obtained by sampling the random noise distribution of the generator, $D\left(G\left(z^{i}\right)\right)$ is the output of the discriminator for the data produced by the generator, $x^{i}$ are the number of real data samples, and $D\left(x^{i}\right)$ is the output of the discriminator for the data produced by the generator.

**Figure 7:**
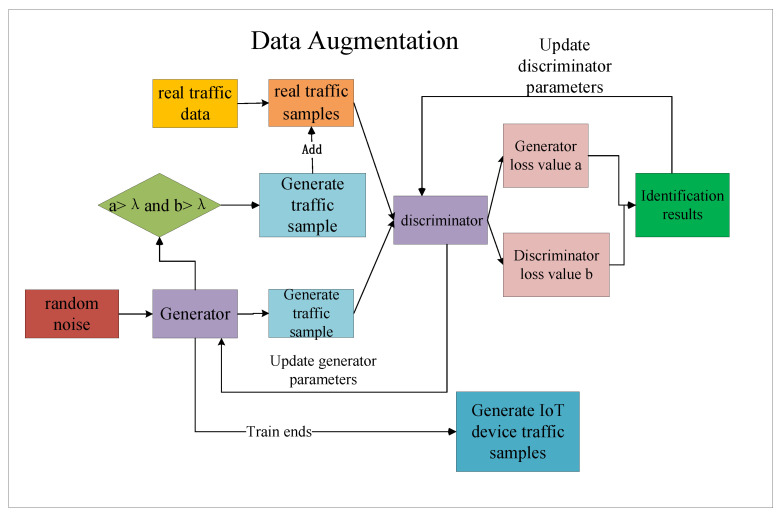
The data augmentation module.

In summary, the NTGAN algorithm, i.e., the data enhancement module, as shown in Figure 7, was proposed to address the issue of insufficient sample numbers for certain devices in the IoT terminal device traffic sample dataset.

The standard GAN has difficulties in generating IoT terminal device traffic sample data for low traffic sample devices. Because the GAN does not learn sufficient number of traffic features from the IoT terminal devices, the realism of the generated samples needs improvement to accurately mimic the actual ones. To solve this issue, in this work, the standard GAN method was modified.

First, for IoT terminal devices with different sample numbers (i.e., devices with low sample numbers), the loss value thresholds, λ, of the generator and the discriminator were set separately. During NTGAN training, when the loss values of both the generator and the discriminator were less than the thresholds, traffic samples generated by the generator were merged with the real traffic samples so that the NTGAN can learn more information about the traffic features of these IoT terminal devices. This threshold mechanism helps ensure that only high-quality, stable synthetic samples are added to the training dataset, preventing performance degradation due to noisy or unrepresentative data. Finally, after training on a certain number of batches, the generator produced traffic samples for IoT terminal devices to balance the number of samples in the dataset. This data augmentation approach increased the quality of the generated samples by introducing thresholds, thus enhancing the ability of the model to learn the traffic features of IoT terminal devices with a small number of samples. Moreover, this strategy reduces the risk of overfitting to the majority class by offering more diverse and representative training data for minority-class devices.

The NTGAN training process is outlined in detail in Algorithm 2. Initially, the raw traffic data from IoT terminal devices, which is in pcap format, undergoes preprocessing to convert it into a set of traffic sample files in png format. These png files serve as input data for the NTGAN module. The primary role of the NTGAN module is to address class imbalance in the dataset by generating additional synthetic samples specifically for the IoT terminal devices that are under-represented. By creating these new samples, NTGAN effectively augments the dataset, ensuring a more balanced distribution of classes. In doing so, the module improves the representational fairness across all device categories, thereby contributing to a more robust and generalizable identification model.

At the conclusion of the process, the output is a comprehensive IoT terminal device traffic dataset, stored as an image file named File_png, which encapsulates the balanced traffic data for subsequent analysis or model training. Let the loss value of the generator be β and the loss value of discriminator be α.
**Algorithm 2:** NTGAN Algorithm    **Input**: Files, Batch_size, Epoch, loss_function, class_count, λ generate_count    **Output**: File_png  **1****for** *each file_class in Files* **do**  **2**        **if** file_class_count<class_count **then**  **3**              train_image=file_class  **4**              true_samples=train_image  **5**              *Set generator_model and discriminator_model parameters, Epoch, Batch_size, etc.*  **6**              **for** *each epoch in (1,Epoch)* **do**  **7**                     **for** *each data in train_image* **do**  **8**                            Generate 100 dimensions of random noise  **9**                            Input noise into generator_model to generate false samples **10**                            Input true samples and false samples to the discriminator_model**11**                            Calculate generator_model loss value β with equation (2)**12**                            Calculate discriminator_model loss value α with equation (3)**13**                            Update generator and discriminator parameters based on their respective loss values**14**                            **if** *$\alpha <\lambda_{loss}$ and $\beta <\lambda_{loss}$* **then****15**                                   Use the generator_model to generate 1000 samples and append to true_samples**16**                            **end****17**                      **end****18**               **end****19**               Use the generator_model to generate generate_count samples and append to Files**20**         **end****21** **end****22** **return** File_png

### 3.3. ResNet18-BiLSTM Model for IoT Terminal Device Identification

**Figure 8:**
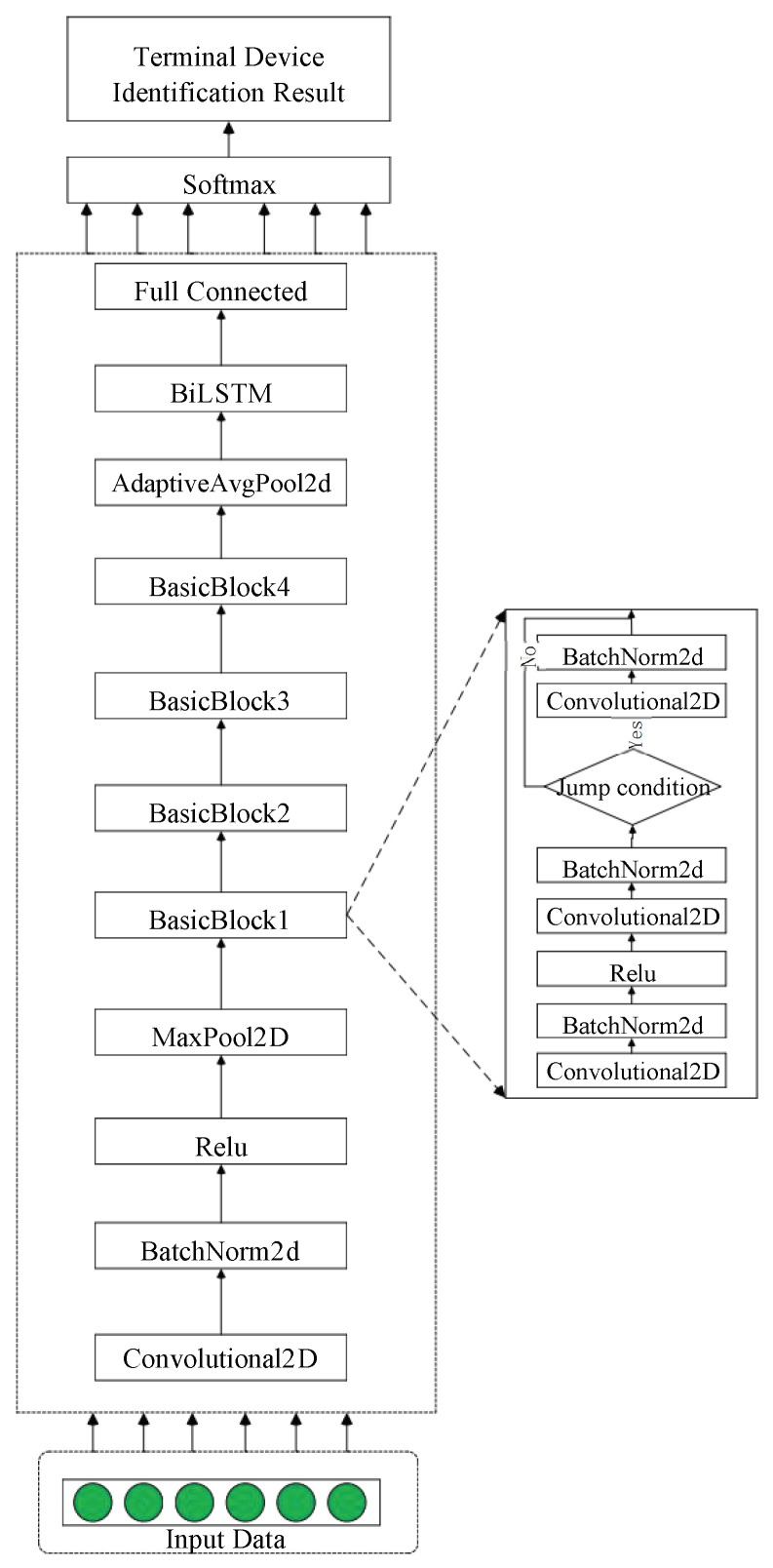
The ResNet18-BiLSTM neural network architecture diagram.

The raw IoT terminal device traffic features input into the Softmax classifier (a common multi-class classification function) cannot efficiently and accurately identify IoT terminal devices. To address this problem, a neural network model must be employed to mine the deep features in the raw IoT terminal device traffic. In this study, a ResNet18-BiLSTM IoT terminal device identification model is proposed. Figure 8 shows the neural network structure of the ResNet18-BiLSTM model. First, the ResNet18 structure in ResNet18-BiLSTM model is used to deeply mine the spatial features in IoT terminal device traffic. Subsequently, the BiLSTM structure is used to extract the temporal features in the IoT terminal device traffic. Finally, the Softmax classifier is used to identify the IoT terminal devices.

To fully bridge the spatial feature representation of ResNet18 and the sequential modeling of BiLSTM, the output tensor of the ResNet18 backbone, with a shape of (Batch,512,7,7), is first processed by an AdaptiveAvgPool2d layer. This pooling operation effectively downsamples the spatial dimensions to (2,2) to optimize computational efficiency and control the sequence length. Subsequently, the pooled tensor is reshaped by flattening the spatial dimensions into a sequence, resulting in a tensor shape of (Batch,4,512), which is then fed into the BiLSTM layer. After the recurrent processing of the sequence, a generic flatten operation is applied to convert the sequential hidden states into a single-dimensional feature vector, which is finally mapped to the class probabilities via the densely connected layer.

#### 3.3.1. ResNet18

After the raw network traffic data generated by the IoT terminal devices were preprocessed and enhanced, the obtained data was stored as a grayscale image. In this study, the ResNet18 model was used to fully exploit the spatial features in the traffic of the IoT terminal devices. ResNet18 is a deep convolution-based neural network model. Unlike the traditional convolutional neural network structure, the ResNet18 mainly consists of an input layer, a convolutional layer, a pooling layer, a global average pooling layer, a residual block, and a fully connected layer. In this study, the input layer in the ResNet18 provided the raw IoT terminal device traffic data. The convolutional and pooling layers were used to extract low level features from the IoT terminal device traffic. The global average pooling layer was located at the top of the ResNet18 and was used to reduce the dimensions of the feature maps by converting them into vectors.

**Figure 9:**
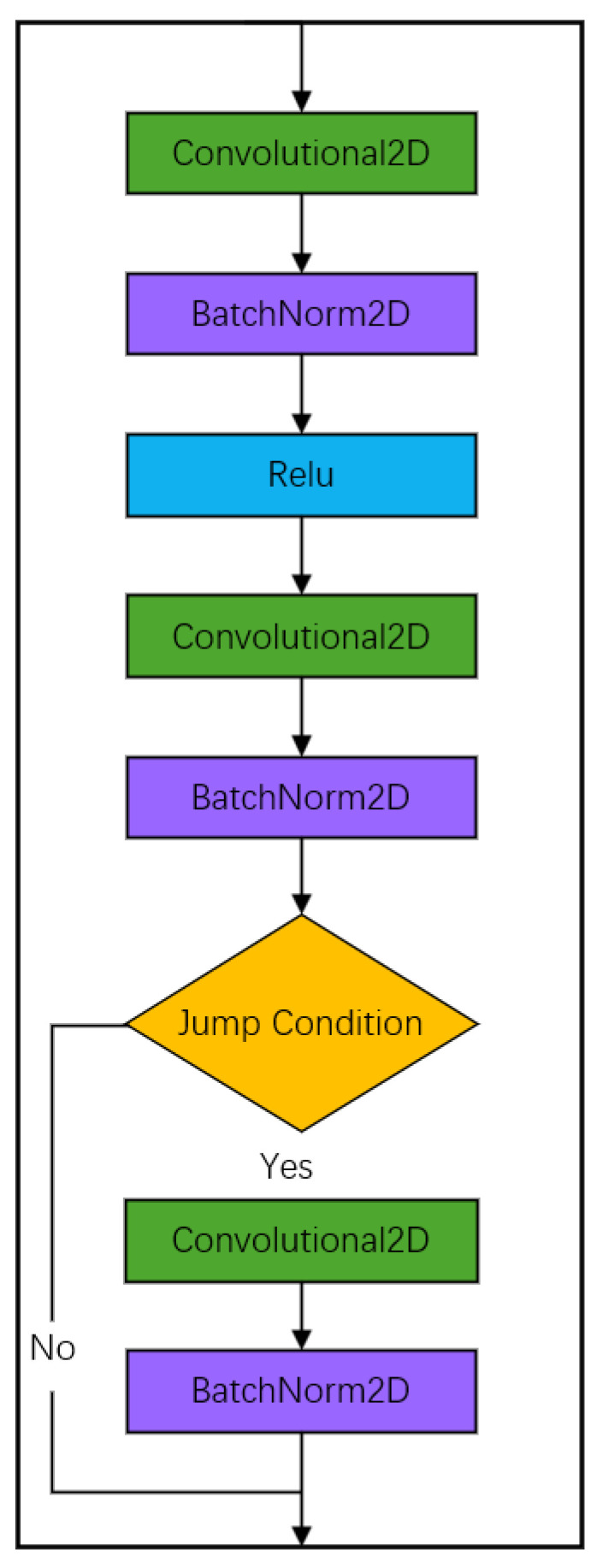
The residual block structure in a ResNet18 network.

In addition, the ResNet18 introduces a residual block structure to solve training problems such as gradient disappearance and explosion. Residual blocks are mainly of two types: basic blocks and bottleneck blocks. Basic blocks consist of convolutional layers, batch normalization layers, rectified linear unit (ReLU) activation functions, and jump connection structures. The convolutional kernel size of the convolutional layer is usually 3×3 and is often used with a bulk normalization layer and ReLU activation function. The jump-junction structure is implemented by adding the inputs directly to the outputs and is used to retain more low-level features, helping to avoid the gradient vanishing problem of the ResNet18. The basic block is commonly used in shallower ResNet18 models. The bottleneck block usually consists of 1×1, 3×3, and 1×1 convolutional layers, a batch normalization layer, an ReLU activation function, and a jump-junction structure. This structure reduces the number of parameters while increasing the depth of the neural network and is commonly used in ResNet18 models with deeper networks. In this study, the basic block structure in the residual block was used to mine the spatial features at a deeper level of the IoT terminal device traffic. Figure 9 shows the residual block network structure used in this work. Compared with the standard convolutional neural network, the ResNet18 can learn spatial features at a deeper level in the IoT terminal device traffic and improve the accuracy of the IoT terminal device identification model.

#### 3.3.2. BiLSTM Neural Network

LSTM is type of recurrent neural network. The difference between LSTM and a standard recurrent neural network is that LSTM avoids the problems of gradient explosion and gradient vanishing by storing cell states and a gate structure. It performs well in extracting temporal features from sequential data. BiLSTM is a bidirectional recurrent neural network. Compared with LSTM, BiLSTM can extract the bidirectional time series features of IoT terminal device traffic. The BiLSTM structure consists of input, forget, and output gates, memory cells, and hidden states. At time *t*, the forget gate ($f_{t}$), input gate ($i_{t}$), and output gate ($o_{t}$) are calculated as shown in Equations (4)–(6), respectively:

(4) $$f_{t}=\sigma \left(W_{f}\cdot \left[h_{t-1},x_{t}\right]+b_{f}\right)$$

(5) $$i_{t}=\sigma \left(W_{i}\cdot \left[h_{t-1},x_{t}\right]+b_{i}\right)$$

(6) $$o_{t}=\sigma \left(W_{o}\cdot \left[h_{t-1},x_{t}\right]+b_{o}\right)$$

where $W_{f}$, $W_{i}$, and $W_{o}$ and $b_{f}$, $b_{i}$, and $b_{o}$ are the weight matrices and neural network bias values of the forget, input, and output gates, respectively; $h_{t-1}$ denotes the hidden state at time t−1; σ is the activation function; and $x_{t}$ denotes the input traffic feature information at time *t*.

At time *t*, multiplying the forget gate value, t−1, by the previous cell state, $C_{t-1}$, and adding to the value of cell state, ${\tilde{C}}_{t}$, multiplied by the memory gate value, we obtain the current cell state, $C_{t}$, as shown in Equations (7) and (8).

(7) $${\tilde{C}}_{t}=\tanh\left(W_{c}\left[h_{t-1},x_{t}\right]+b_{c}\right)$$

(8) $$C_{t}=f_{t}\times C_{t-1}+i_{t}\times {\tilde{C}}_{t}$$

The unimportant IoT terminal device traffic information transmitted by the unit at time t−1 is filtered out by the forget gate. Subsequently, the important IoT terminal device traffic information at time *t* is extracted by the input gate. Finally, the hidden state, $h_{t}$, at time *t* is calculated by the output gate, as shown in Equation (9).

(9) $$h_{t}=o_{t}\times \tanh\left(C_{t}\right)$$

BiLSTM combines the forward LSTM hidden states, ${\vec{h}}_{t}$, and reverse LSTM hidden states, ${\vec{h}}_{t}$, to generate a new hidden state, $h_{t}$, which is computed as shown in Equation (10).

(10) $$h_{t}=\left[{\vec{h}}_{t};{\overset{\leftarrow }{h}}_{t}\right]$$

#### 3.3.3. ResNet18-BiLSTM

This study integrated ResNet18 and BiLSTM neural networks to capture temporal and spatial features of IoT terminal device traffic, proposing the ResNet18-BiLSTM identification model. The pseudo-code for this model is shown in Algorithm 3.

Initially, raw IoT traffic samples were processed with the NTGAN module to generate additional samples for under-represented devices, mitigating data imbalance and yielding the standardized dataset, File_png, in grayscale image format. This dataset was randomly separated into training ($X_{train}$), validation ($X_{val}$), and test sets ($X_{test}$) in a 6:2:2 ratio. Model training followed by setting hyperparameters and iteratively processing $X_{train}$ and $X_{val}$ batches, with $X_{train}$ used for training and $X_{val}$ for validation. Ultimately, the trained IoT device identification model *M* was obtained.
**Algorithm 3:** ResNet18-BiLSTM Model    **Input:** $X_{train}$, $X_{val}$, Epoch, dropout, Loss_function, Batch_size    **Output:** *M*  **1**Set model parameters: Epoch, Batch_size, learning_rate, etc.;  **2****for** *each epoch in (1, Epoch)* **do**  **3**        $X=X_{train}$;  **4**        **for** *each batch_size in X* **do**  **5**                **for** *each data point in batch_size* **do**  **6**                        Compute convolution with 7 filters  **7**                        Use Batch Normalization;  **8**                        Run through Max Pooling layer;  **9**                        Run through make_layer 1 4;**10**                        Run through AdaptiveAvgPool2d layer;**11**                        Reshape spatial dimensions into a sequence length;**12**                        Use Equation (4) to calculate the forgetting gate at time *t*;**13**                        Use Equation (5) to calculate the input gate at time *t*;**14**                        Use Equation (6) to calculate the output gate at time *t*;**15**                        Use Equations (7) and (8) to calculate the unit state at time *t*;**16**                        Use Equations (9) and (10) to calculate the hidden state at time *t*;**17**                        Run through flatten layer;**18**                        Run through a densely connected layer;**19**                **end****20**        **end****21**        ResNet18-BiLSTM($X_{val}$) *// Evaluate validation set using the ResNet18-BiLSTM model*;**22** **end****23** Save *M // Save the trained IoT device recognition model*;**24** **return** *M*

### 3.4. Introduction to IoTDI-ImbS

We propose the IoTDI-ImbS scheme, which integrates data preprocessing, the NTGAN algorithm, and the ResNet18-BiLSTM model. The process operates as follows: First, the input raw IoT terminal device traffic sample set (*T*) in pcap format undergoes data preprocessing via Algorithm 1, yielding grayscale image sample files. To ensure a rigorous evaluation and strictly prevent data leakage, this grayscale image set is first subjected to stratified partitioning into a training set ($X_{train}$), a validation set ($X_{val}$), and a test set ($X_{test}$) with a ratio of 6:2:2. Subsequently, the NTGAN module (Algorithm 2) is applied exclusively to the training set ($X_{train}$) to synthesize high-quality samples for minority classes, resulting in an augmented and balanced training set ${X^{\prime }}_{train}$. During this stage, the test set ($X_{test}$) remains completely untouched and physically isolated to simulate a real-world unseen environment. Next, Algorithm 3 utilizes the augmented ${X^{\prime }}_{train}$ and the original $X_{val}$ to train the ResNet18-BiLSTM model, yielding the IoT device recognition model *M*. Finally, the identification performance is evaluated by testing *M* on the original, unseen test set ($X_{test}$). The refined pseudo-code for the integrated IoTDI-ImbS process is provided in Algorithm 4.
**Algorithm 4:** IoTDI-ImbS Method**Input:** *T* // Raw IoT terminal device traffic sample set (format: pcap)**Output:** DI_R // IoT recognition results**1** Files←DPP(T) *// Call Algorithm 1*;**2** $X_{train},X_{val},X_{test}\leftarrow \mathrm{split}\left(Files\right)$ *// Randomly split Files into training, validation, and testing sets without replacement*;**3** $X_{train}\leftarrow NTGAN\left(X_{train}\right)$ *// Call Algorithm 2*;**4** $M\leftarrow ResNet18-BiLSTM(X_{train},X_{val})$ *// Input training and validation sets*;**5** $\mathrm{Input}X_{test}\leftarrow M$;**6** $DI\text{\_}R\leftarrow softmax(X_{i},X_{test})$ *// Evaluate and test*;**7** **return** DI_R

## 4. Experimental Results and Analysis

### 4.1. Experiment Objectives

The primary objective of this experimental study is to evaluate the effectiveness and robustness of the proposed IoTDI-ImbS method in identifying IoT terminal devices under conditions of imbalanced traffic data. Specifically, the experiments aim to verify the following aspects:

- Payload feature selection improves feature representation. We assess whether selecting payload information from network traffic as the raw feature leads to more discriminative representations of device behavior.
- The NTGAN data augmentation module effectively alleviates sample imbalance. We evaluate the ability of NTGAN to generate realistic synthetic traffic samples for under-represented device classes and examine its impact on recognition accuracy.
- The ResNet18-BiLSTM hybrid model enhances spatial-temporal feature extraction. We analyze whether combining residual convolutional layers and recurrent temporal modeling improves classification performance, especially for complex or ambiguous traffic patterns.
- IoTDI-ImbS outperforms traditional and deep learning baselines. We compare the proposed method with several state-of-the-art baselines in terms of multiple metrics (accuracy, precision, recall, and F1-score) to demonstrate its superiority.
- Recognition improvements are consistent across both large- and small-scale datasets. We validate the generalizability of the method by testing on two datasets of different sizes and traffic distributions.

These objectives collectively assess not only the performance of the model under balanced and imbalanced conditions, but also the contribution of each module to the overall design. The experiments are structured to progressively analyze each component and their synergies, as well as the method’s practical applicability.

### 4.2. Datasets and Preprocessing

In our experiments, two widely used benchmarks are employed to evaluate the proposed IoTDI-ImbS model. While the IoT Sentinel dataset exhibits higher category diversity with 27 distinct device classes, the UNSW dataset provides a significantly larger data volume and a more pronounced class imbalance across its 22 device categories. Specifically, the UNSW dataset contains millions of packets, offering a more comprehensive representation of long-term traffic behavior, whereas the IoT Sentinel dataset focuses on the initial setup phase of a broader variety of devices.

The IoT Sentinel dataset represents a resource-constrained environment, containing limited traffic samples for most IoT devices. It is suitable for evaluating the method’s capability to identify devices with minimal data. In contrast, the UNSW dataset includes extensive traffic data from a broader range of IoT devices, offering a rich and diverse testbed for large-scale recognition tasks and sample imbalance analysis.

The two datasets together cover 49 different IoT terminal devices, including smart cameras, smart plugs, motion sensors, door sensors, and other common household or industrial IoT equipment. Each dataset was collected through packet capture over defined time periods, with necessary anonymization and preprocessing applied to ensure consistency and realism.

Table 1 summarizes the number of samples for each device in both datasets. Notably, many devices in the IoT Sentinel dataset have fewer than 1000 samples, whereas the UNSW dataset includes devices with over 100,000 samples. This discrepancy underscores the data imbalance problem and provides a strong basis for evaluating the effectiveness of the NTGAN module.

Furthermore, the selection of these two datasets allows for a multi-dimensional assessment of the IoTDI-ImbS method. By testing on both the sparse IoT Sentinel data and the voluminous UNSW data, we can verify whether the proposed framework maintains high recognition accuracy while mitigating the risk of overfitting in data-scarce classes.

### 4.3. Evaluation Metrics

To quantitatively evaluate the performance of IoT device identification, we adopt four widely used classification metrics: accuracy, precision, recall, and F1-score. Each of these metrics captures a different aspect of model performance:

- Accuracy reflects the overall correctness of the model across all classes.
- Precision focuses on how many of the predicted positive instances are actually correct, which is particularly useful when false positives are costly.
- Recall measures how many of the actual positive instances are successfully detected and is crucial when identifying all relevant classes is important.
- F1-score is the harmonic mean of precision and recall, providing a balanced measure when trade-offs between the two are necessary.

The definitions of the metrics are provided as follows:

(11) $$\mathrm{Accuracy}=\frac{1}{N}\sum_{i=1}^{N}\frac{{\mathrm{TP}}_{i}+{\mathrm{TN}}_{i}}{{\mathrm{TP}}_{i}+{\mathrm{TN}}_{i}+{\mathrm{FP}}_{i}+{\mathrm{FN}}_{i}}$$

(12) $$\mathrm{Precision}=\frac{1}{N}\sum_{i=1}^{N}\frac{{\mathrm{TP}}_{i}}{{\mathrm{TP}}_{i}+{\mathrm{FP}}_{i}}$$

(13) $$\mathrm{Recall}=\frac{1}{N}\sum_{i=1}^{N}\frac{{\mathrm{TP}}_{i}}{{\mathrm{TP}}_{i}+{\mathrm{FN}}_{i}}$$

(14) $$F1\text{-}\mathrm{score}=\frac{2\times \mathrm{Precision}\times \mathrm{Recall}}{\mathrm{Precision}\;+\;\mathrm{Recall}}$$

Here, TP (true positives), TN (true negatives), FP (false positives), and FN (false negatives) represent the classification results across different device classes, let *N* denote the total number of distinct target classes. In addition to these metrics, we utilize confusion matrices to visualize and analyze misclassification patterns, which provide intuitive insights into class-wise prediction errors, especially under sample imbalance conditions.

### 4.4. Experimental Setup

All experiments were conducted on a desktop platform equipped with an Intel Core i7-9700 CPU, 32 GB RAM, and an NVIDIA GeForce GTX 1660 SUPER GPU, running Windows 10. The implementation environment includes Python 3.8 with support from both TensorFlow 2.10.0 and PyTorch 1.11.0. Detailed configuration is shown in Table 2.

The proposed IoTDI-ImbS framework was trained using a batch size of 32 over 50 epochs. We employed the AdamW (Adaptive Moment Estimation with Weight Decay) optimizer with a learning rate of 0.0001. The loss function used for training was categorical cross-entropy (a common loss function for multi-class classification). The architectural details of the ResNet18-BiLSTM model are summarized in Table 3.

In addition, the NTGAN data augmentation module was used to generate additional synthetic traffic samples for under-represented device classes. The network structure of NTGAN includes four dense layers in both the generator and discriminator, along with LeakyReLU activation and dropout regularization. A loss threshold mechanism was introduced to control the quality of generated samples. The full layer specifications of NTGAN are provided in Table 4.

### 4.5. Effectiveness of NTGAN Data Augmentation

To evaluate the impact of the NTGAN module on addressing data imbalance, we conducted a series of comparative experiments on both the UNSW and IoT Sentinel datasets, under two conditions: with and without NTGAN-generated augmented samples. Performance was comprehensively assessed using three commonly accepted classification metrics—precision, recall, and F1-score—across all IoT device categories. These metrics reflect the classifier’s ability to correctly identify devices, avoid false positives, and achieve a balanced trade-off between completeness and correctness.

**Figure 10:**
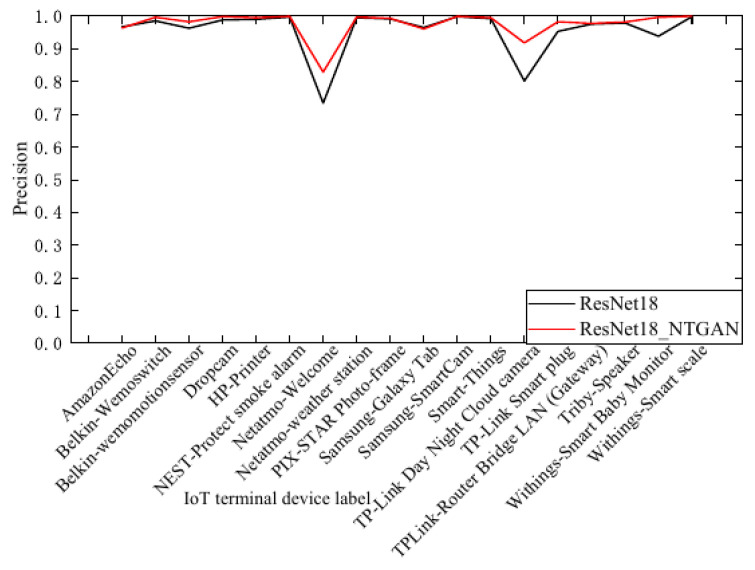
The precision of NTGAN on UNSW dataset.

**Figure 11:**
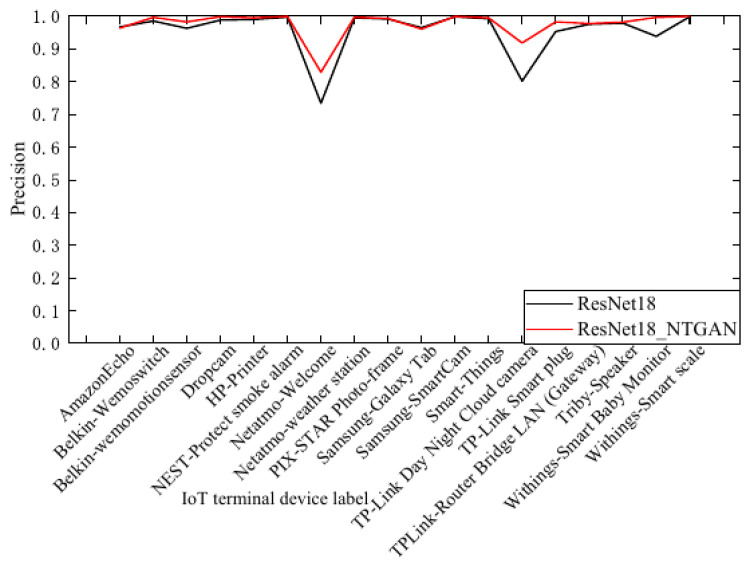
The recall of NTGAN on UNSW dataset.

**Figure 12:**
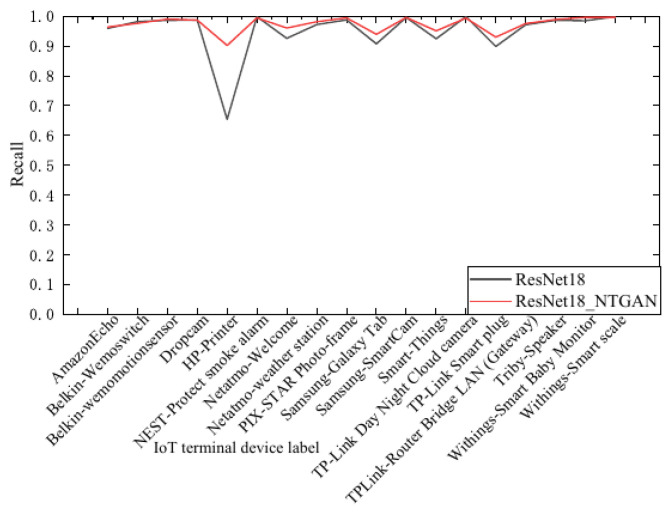
The f1-score of NTGAN on UNSW dataset.

Figure 10, Figure 11 and Figure 12 illustrates the experimental results obtained on the UNSW dataset. The integration of the NTGAN module yielded notable improvements in the overall performance of the IoT device recognition model. Specifically, the precision increased by approximately 2.0%, recall by 2.2%, and F1-score by 2.4% when compared to the baseline model without NTGAN. These gains, while seemingly modest in average, reflect significant improvements for specific under-represented device types. For instance, IoT devices such as Netatmo Welcome, TP-Link Day Night Cloud Camera, and HP Printer—which originally suffered from limited sample sizes—showed particularly marked improvements. Taking the HP Printer as an example, its F1-score increased by a substantial 15.9%, indicating that the model became much more accurate and consistent in recognizing its traffic patterns after the NTGAN-based data augmentation.

**Figure 13:**
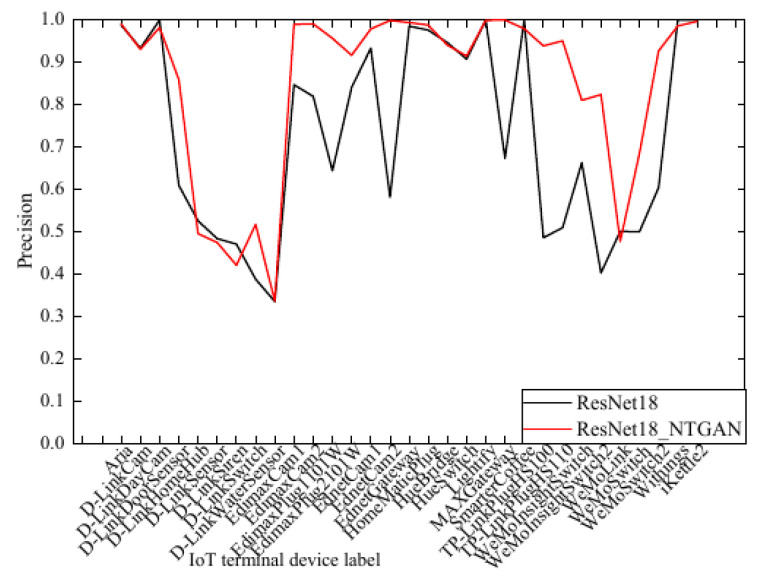
The precision of NTGAN on IoT Sentinel dataset.

**Figure 14:**
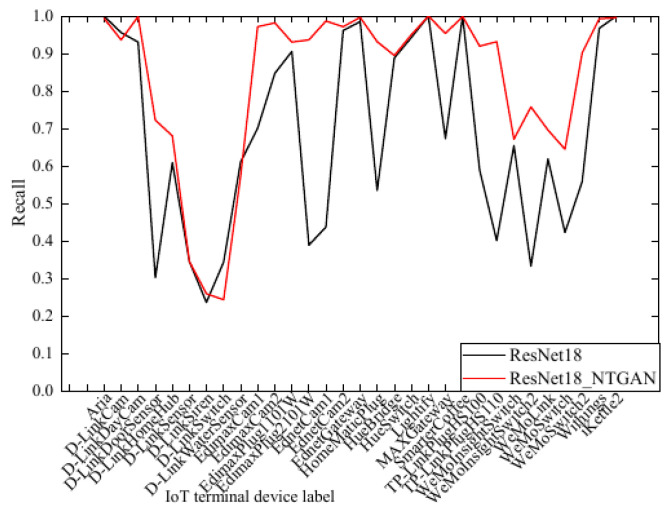
The recall of NTGAN on IoT Sentinel dataset.

**Figure 15:**
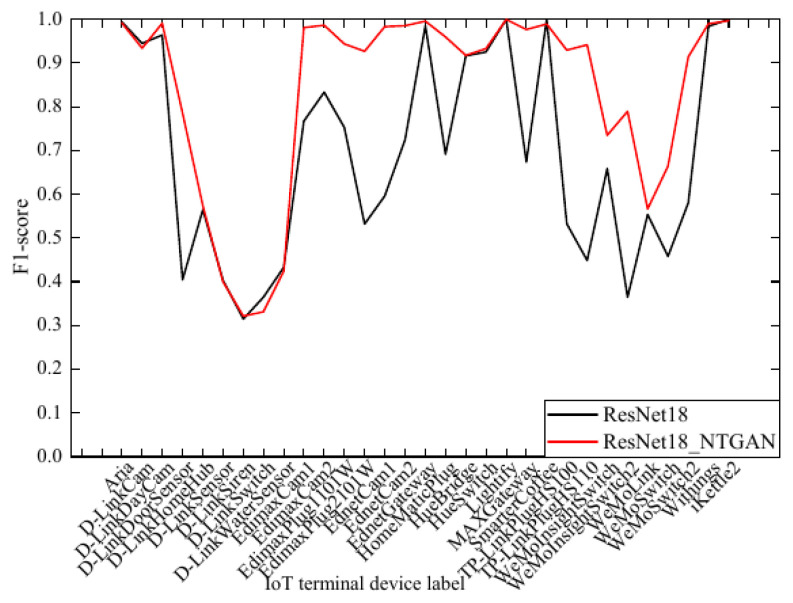
The f1-score of NTGAN on IoT Sentinel dataset.

The benefits of NTGAN are even more pronounced in the IoT Sentinel dataset, which is characterized by extreme sample imbalance and scarcity of training data for most device categories. As shown in Figure 13, Figure 14 and Figure 15, the addition of NTGAN to the training process led to an overall 14.5% increase in the F1-score of the recognition model. Out of the 31 IoT devices present in this dataset, 21 devices achieved a recognition accuracy exceeding 90%, demonstrating the model’s improved generalization ability. Furthermore, certain devices such as EdimaxPlug1101W, TP-LinkPlug HS100, and WeMoSwitch2 displayed drastic improvements across all three metrics. In several cases, the F1-score of individual devices improved by more than 30%, indicating a major enhancement in recognizing rare or low-traffic IoT devices.

These performance gains can be attributed to two key mechanisms introduced by the NTGAN framework. First, NTGAN effectively expands the coverage of the feature space for under-represented classes. By generating synthetic traffic samples that mirror the real distribution, it improves the diversity of the training data and enables the classifier to better learn the boundaries between classes. Second, the introduction of a loss threshold mechanism ensures that only high-quality, semantically consistent synthetic samples are retained, thereby avoiding the introduction of noise or artifacts that could harm the model’s convergence or decision boundaries. This dual design enables the model not only to benefit from augmented data but also to retain stability and robustness during training.

In conclusion, the experimental results clearly demonstrate that the NTGAN module plays a crucial role in mitigating the sample imbalance problem inherent in IoT device recognition tasks. By synthetically enriching minority classes and controlling sample quality, NTGAN substantially improves recognition accuracy, particularly for devices with scarce data. The module not only compensates for the statistical limitations of imbalanced datasets but also helps the model generalize better to unseen traffic patterns. These results affirm that NTGAN is not merely a supplementary component but a core enabler of the overall IoTDI-ImbS architecture, significantly enhancing its real-world applicability and deployment potential in diverse IoT environments. Its integration ensures that IoT device identification remains effective and stable even in scenarios with dynamic, heterogeneous, and data-sparse operating conditions.

### 4.6. Ablation Study

To investigate the contribution of individual components in the IoTDI-ImbS architecture, we conducted ablation experiments comparing three model variants:

- ResNet18-only: baseline spatial feature extractor using convolutional residual blocks.
- ResNet18-BiLSTM: adds a BiLSTM layer to capture temporal dependencies across packet sequences.
- ResNet18-BiLSTM-AM: introduces an attention mechanism to emphasize important features after spatial-temporal extraction.

**Figure 16:**
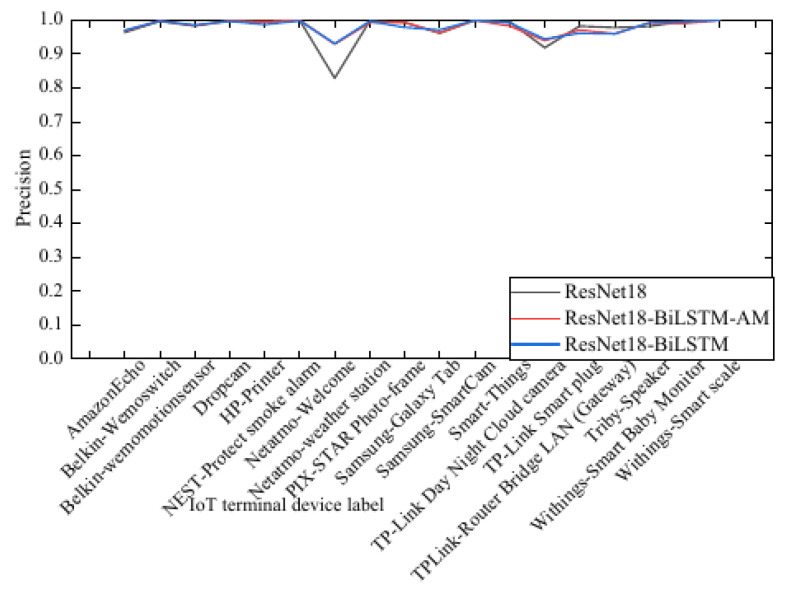
Precision of ablation experiments on UNSW Dataset.

**Figure 17:**
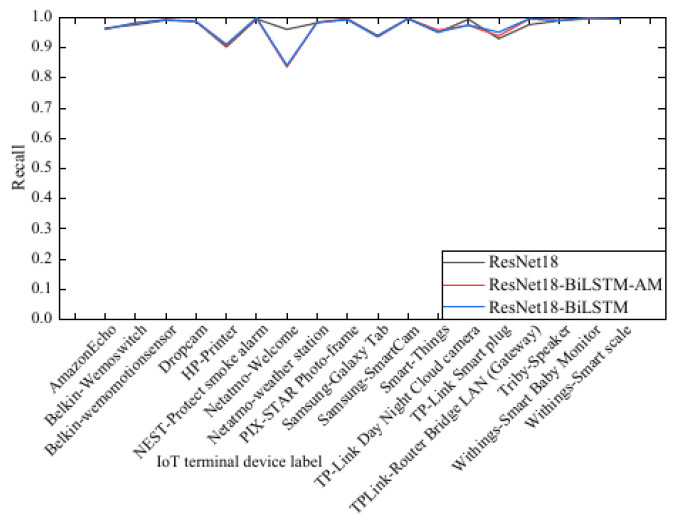
Recall of ablation experiments on UNSW Dataset.

**Figure 18:**
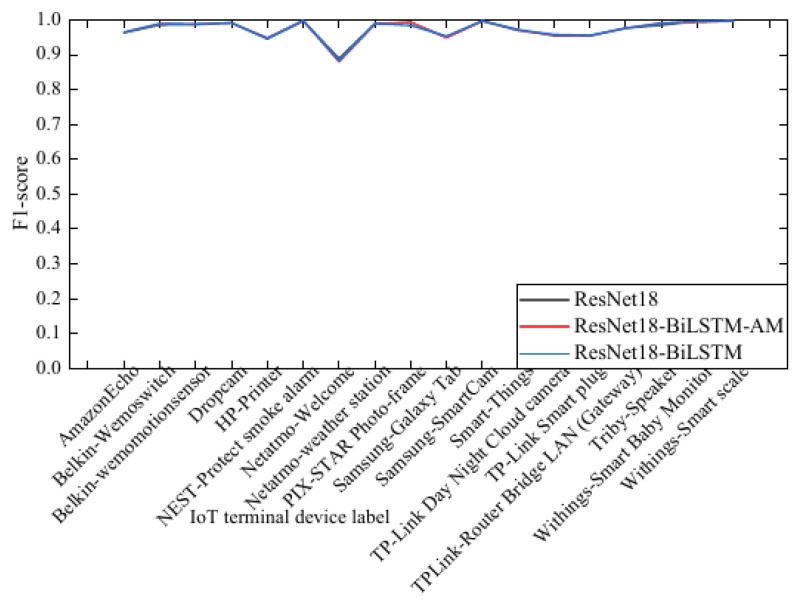
F1-score of ablation experiments on UNSW Dataset.

**Figure 19:**
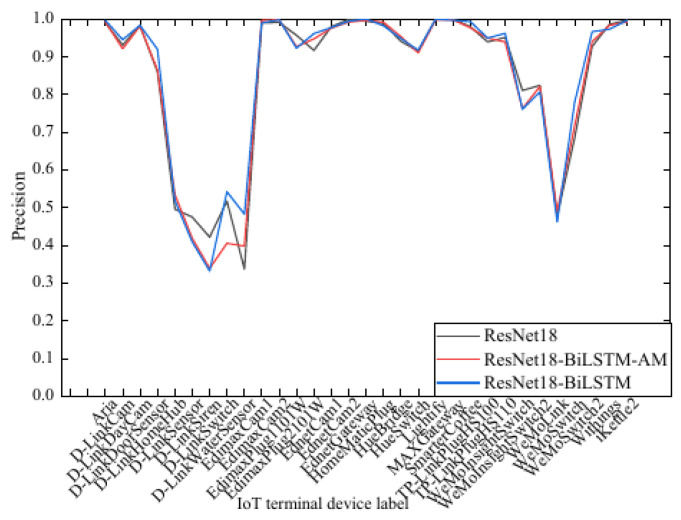
Precision of ablation experiments on IoT Sentinel Dataset.

**Figure 20:**
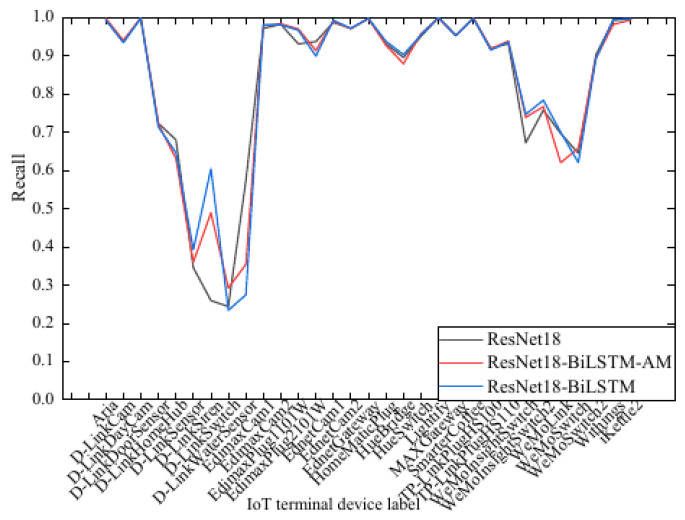
Recall of ablation experiments on IoT Sentinel Dataset.

**Figure 21:**
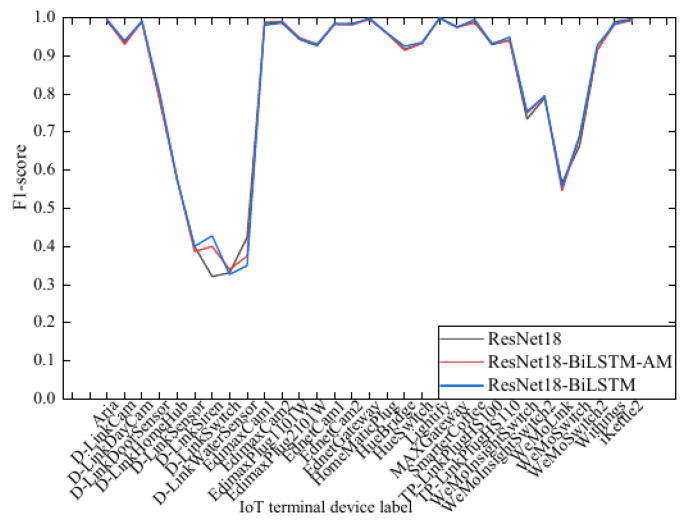
F1-score of ablation experiments on IoT Sentinel Dataset.

The experiments were performed on both the UNSW and IoT Sentinel dataset. The results are presented in Figure 16, Figure 17, Figure 18, Figure 19, Figure 20 and Figure 21 showing the comparison of precision, recall, and F1-score across all device types.

On the UNSW dataset (The ResNet18-BiLSTM variant outperformed the ResNet18-only model, improving F1-score by more than 2% on average. This confirms that modeling temporal correlations in network traffic is critical for improving recognition accuracy, especially when packet order carries semantic meaning.

On the IoT Sentinel dataset (Figure 19, Figure 20 and Figure 21), which contains fewer samples per device, a similar trend was observed. The ResNet18-BiLSTM model consistently outperformed other variants, particularly in recall and F1-score. Although the attention-enhanced model performed better for individual devices like D-LinkWaterSensor, its average performance was slightly inferior due to overfitting to local features.

### 4.7. Validation of the Validity of the IoTDI-ImbS Methodology

These results suggest that while BiLSTM plays a vital role in capturing temporal dynamics in network traffic, the attention module does not universally improve performance and may require further tuning or selective application.

In summary, the ResNet18-BiLSTM structure provides a balanced and effective architecture for extracting spatial-temporal features from IoT traffic, validating its inclusion as the core of the proposed IoTDI-ImbS framework.

### 4.8. Model Performance and Confusion Analysis

**Figure 22:**
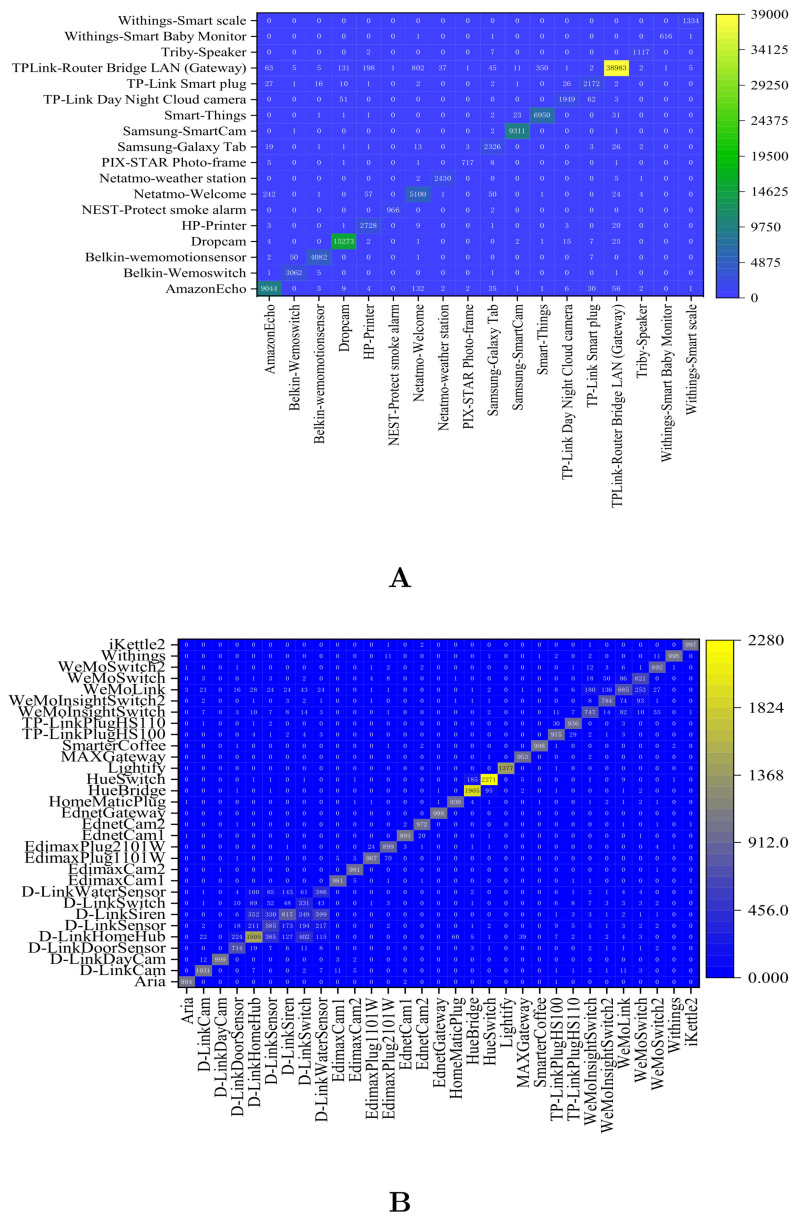
Ablation experimental results for IoT Sentinel dataset. (**A**) Confusion matrix of experimental results for UNSW dataset. (**B**) Confusion matrix of experimental results for IoT Sentinel dataset.

**Figure 23:**
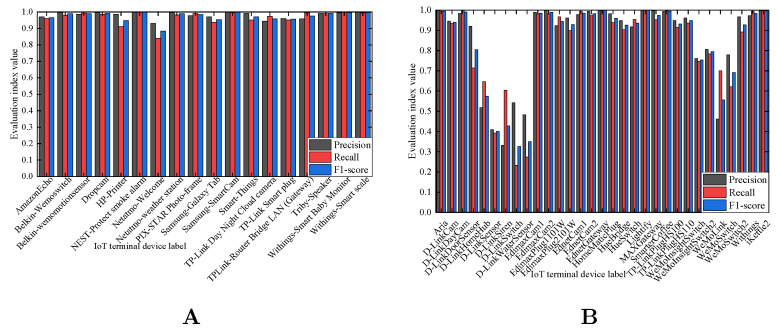
Classification effect of IoT terminal devices in different datasets. (**A**) Classification effect of IoT terminal devices in UNSW dataset. (**B**) Classification effect of IoT terminal devices in IoT Sentinel dataset.

To further assess the practical effectiveness of the IoTDI-ImbS method, we evaluate its classification performance across all 49 IoT device types using confusion matrices. Figure 22 presents confusion matrices for both the UNSW and IoT Sentinel dataset, while Figure 23 illustrates per-device classification accuracy.

The IoTDI-ImbS model successfully identified all 49 device types in the two datasets, achieving an overall accuracy of 97.4% and an F1-score of 0.974 on the UNSW dataset. Notably, 11 devices exhibited both recall and F1-scores above 0.98. On the smaller and more imbalanced IoT Sentinel dataset, the method still maintained a strong F1-score of 0.839, with 21 devices recognized at an accuracy above 90%.

Despite the overall strong performance, some misclassification patterns were observed. In particular, Amazon Echo and Netatmo Welcome were frequently confused with each other. This can be attributed to their overlapping traffic patterns—both are multimedia IoT devices with similar session behaviors and packet frequency. Similarly, TP-Link Router Bridge LAN (Gateway) samples were occasionally misclassified due to the device’s large sample size and broad traffic feature coverage, which could introduce overlaps with other device classes.

On the IoT Sentinel dataset, misclassifications were more common among D-Link family devices such as D-LinkWaterSensor, D-LinkSwitch, and D-LinkHomeHub. These devices likely share similar protocol stacks and packet structures due to being from the same manufacturer, which reduces inter-class variance and poses challenges to discriminative learning. Additionally, the limited number of samples for these devices further weakens the model’s ability to learn distinctive patterns.

Nonetheless, the confusion matrix analysis confirms that the majority of errors are concentrated within a few closely related device pairs. The model demonstrates strong generalization across both datasets, including successful identification of rare-class devices enhanced by NTGAN.

In summary, IoTDI-ImbS achieves robust classification performance in both balanced and imbalanced data conditions, with the confusion matrix analysis revealing that most misclassifications stem from intrinsic traffic similarity rather than model instability. This supports the reliability and practicality of the proposed method in real-world deployment.

### 4.9. Comparison with Baseline Models

To validate the overall efficiency of the IoTDI-ImbS method, we compared its performance against several existing IoT device identification approaches using the UNSW dataset. The comparison includes methods based on sliding window [7], encrypted traffic patterns [8], random forest classification [9], statistical behavior analysis [10], machine learning ensemble [16], two-stage multi-classification [17], and lightweight convolutional neural networks [18].

**Figure 24:**
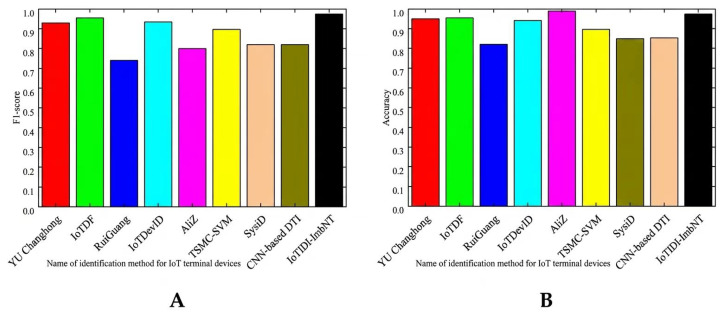
A comparison of multiple evaluation indicators for different IoT terminal device identification methods. (**A**) F1-score and (**B**) accuracy.

As illustrated in Figure 24, IoTDI-ImbS achieves the highest F1-score (0.974) among all evaluated methods, indicating superior balance between precision and recall. Although its accuracy is slightly lower than the method based on two-stage multi-classification [17], its significantly higher F1-score (by 0.174) confirms more consistent performance across both majority and minority classes. This is particularly important in scenarios where class imbalance is prominent.

The superior performance of IoTDI-ImbS can be attributed to three key factors:

- Feature selection strategy: The use of payload data preserves fine-grained device behavior information, providing a richer feature space than statistical summaries or encrypted headers.
- Imbalance-aware augmentation: The NTGAN module effectively balances the training set by synthesizing realistic traffic for rare devices, which enhances classifier robustness.
- Hybrid spatial-temporal modeling: The ResNet18-BiLSTM architecture captures both structural and sequential patterns in traffic, leading to more discriminative representations compared to shallow models or purely CNN-based designs.

Taken together, these design choices enable IoTDI-ImbS to achieve high classification performance across both dominant and under-represented device types. Its advantage is particularly pronounced in real-world environments with highly skewed data distributions, demonstrating its practical value for secure and scalable IoT network deployments.

### 4.10. Summary of Experimental Findings

Based on the experimental results across multiple datasets, model configurations, and evaluation metrics, the following key findings are summarized:

- Payload-based feature selection offers superior representational power. By using raw payload data instead of statistical or header features, the model captures finer-grained behavior characteristics, contributing to higher classification accuracy.
- NTGAN effectively addresses class imbalance. The data augmentation module significantly improves the recognition performance of under-represented devices, with F1-score improvements of up to 30% on minority classes in the IoT Sentinel dataset. The performance improvement is primarily attributed to effective sample balancing.
- The ResNet18-BiLSTM architecture balances spatial and temporal feature extraction. Ablation studies show that combining convolutional and recurrent layers outperforms other variants in both overall and per-class metrics.
- IoTDI-ImbS demonstrates consistent generalization across datasets. The method achieves strong results on both large-scale (UNSW) and small-scale (IoT Sentinel) datasets, indicating its robustness in varied network evaluation scenarios.
- Compared to existing methods, IoTDI-ImbS achieves state-of-the-art performance. It obtains the highest F1-score among all baseline methods, particularly excelling in recognizing devices with few training samples.
- While the NTGAN-based data augmentation and the hybrid ResNet18-BiLSTM training require GPU acceleration, this process is performed offline on a central server or cloud platform. Therefore, the training complexity does not affect the performance of IoT end-devices.
  In conclusion, the IoTDI-ImbS method integrates complementary strategies in feature selection, data balancing, and hybrid modeling, resulting in a scalable and effective solution for IoT device identification under imbalanced conditions.

## 5. Conclusions

The IoTDI-ImbS was proposed in this study to address the issue of unbalanced sample distribution in the network traffic originating from certain IoT terminal devices, which impacts the accuracy of IoT terminal device identification methods. First, the original IoT terminal device traffic files were preprocessed to obtain the raw traffic sample set. Subsequently, to address sample imbalance in the dataset, the raw sample set was input into the NTGAN algorithm for training. The ResNet18-BiLSTM model was then used to extract the temporal and spatial features from the network traffic, forming a device fingerprint. Finally, IoT terminal devices were identified by using a Softmax classifier. Experimental results revealed that the IoTDI-ImbS exhibited improved device identification accuracy when applied to two IoT terminal device datasets of different sizes. However, a limitation of this method is that the identification of unknown IoT terminal devices is not fully considered. Future research will be focused on to the development of a method for identifying unknown IoT terminal devices from the network traffic.

Regarding the practical challenges in IoT environments, such as encrypted and dynamic traffic, IoTDI-ImbS maintains its effectiveness through its structural feature extraction.

- Although encryption hides the semantic meaning of payloads, it does not eliminate the statistical distribution and structural patterns of the byte stream. Our Payload-to-Image conversion captures these high-dimensional distributions (e.g., entropy and recurring patterns) as visual textures, which ResNet18 can identify even without decryption.
- The BiLSTM component is designed to capture temporal dependencies across packet sequences. Even if some packets are lost or the traffic rhythm changes, the model can still rely on the remaining sequential logic and the robust spatial features extracted from individual packets to maintain high identification accuracy.
- By identifying the exact device model, network administrators can cross-reference active devices with known vulnerability databases (e.g., CVE). This allows for proactive risk assessment and the deployment of targeted security patches.
- In large-scale deployments like smart cities or industrial IoT, manual tracking is impossible. Our method enables real-time, automated discovery and inventory of devices, ensuring that unauthorized or rogue devices are immediately flagged.

Furthermore, accurate IoT device identification serves as a foundational building block for broader network security paradigms. By establishing the identity of each connected terminal, administrators can implement fine-grained access control and anomaly detection. For instance, precise device profiles are essential for detecting unintended interactions between heterogeneous devices, which could otherwise lead to systemic vulnerabilities [27]. Moreover, robust identification contributes to the early detection of sophisticated cyberattacks, such as unauthorized lateral movement or device spoofing, thereby enhancing the overall resilience of the IoT ecosystem [28]. Integrating our proposed IoTDI-ImbS scheme into such security frameworks could significantly mitigate the risks associated with the proliferation of unmanaged IoT devices.

## Figures and Tables

**Table 1:** Detailed summary of the experimental dataset.

IoT Sentinel Dataset			UNSW Dataset		
No.	Device Name	Size	No.	Device Name	Size
1	Aria	419	1	Amazon Echo	47,046
2	D-LinkCam	5508	2	Belkin Wemo switch	15,592
3	D-LinkDayCam	862	3	Belkin Wemo motion sensor	6741
4	D-LinkDoor Sensor	2108	4	Dropcam	77,390
5	D-LinkHome Hub	11,642	5	HP Printer	3889
6	D-LinkSensor	7439	6	NEST Protect smoke alarm	4831
7	D-LinkSiren	6757	7	Netatmo Welcome	17,690
8	D-LinkSwitch	7080	8	Netatmo weather station	9250
9	D-LinkWater Sensor	7026	9	PIX-STAR Photo-frame	3611
10	EdimaxCam1	431	10	Samsung Galaxy Tab	12,410
11	EdimaxCam2	288	11	Samsung SmartCam	46,742
12	EdimaxPlug1101W	846	12	Smart Things	22,219
13	EdimaxPlug2101W	741	13	TP-Link Day Night Cloud camera	3418
14	EdimaxCam1	157	14	TP-Link Smart plug	6160
15	EdimaxCam2	124	15	TP-Link Router Bridge LAN	195,884
16	EdnetGateway	690	16	Triby Speaker	5633
17	HomeMaticPlug	768	17	Withings Smart Baby Monitor	1314
18	HueBridge	10,538	18	Withings Smart scale	2977
19	HueSwitch	11,894			
20	iKettle2	80			
21	Lightify	6882			
22	MAXGateway	743			
23	SmarterCoffee	82			
24	TP-LinkPlug HS100	726			
25	TP-LinkPlug HS110	653			
26	WeMoInsightSwitch	3646			
27	WeMoInsightSwitch2	1647			
28	WeMoLink	4889			
29	WeMoSwitch	2920			
30	WeMoSwitch2	802			
31	Withings	612			
Total		99,000	Total		482,797

**Table 2:** Experimental environment parameters.

Parameter	Value
CPU	Intel Core i7-9700
GPU	NVIDIA GeForce GTX 1660 SUPER
Memory (GB)	32
Operating System	Windows 10
Programming Language	Python 3.8
Deep Learning Framework	TensorFlow 2.10.0, PyTorch 1.11.0
Development Tool	PyCharm Community Edition 2021.2.2

**Table 3:** Detailed information of experimental parameters.

Parameter	Value	Activation Function
Conv2D	kernel_size=7, stride=2, padding=3	ReLU
MaxPool2D	kernel_size=3, stride=2, padding=1	—
Basic Block-Conv2D	kernel_size=3, stride=1, padding=1	ReLU
Basic Block-Skip Connection-Conv	kernel_size=3, stride=1, padding=1	ReLU
Basic Block-Conv2D	kernel_size=1, stride=1	ReLU
BiLSTM	neurons=128	Tanh
Batch Size	32	—
Epochs	50	—
Learning Rate	0.0001	—
Optimizer	AdamW	—
Loss Function	Categorical Crossentropy	—

**Table 4:** NTGAN network parameters.

Generator Network		Discriminator Network	
Layer	Output Shape	Layer	Output Shape
Dense_1 (Dense)	(None, 256)	Dense_1 (Dense)	(None, 53×53)
LeakyReLU	(None, 256)	LeakyReLU	(None, 53×53)
BatchNormalization	(None, 256)	Dropout	(None, 53×53)
Dense_2 (Dense)	(None, 512)	Dense_2 (Dense)	(None, 1024)
LeakyReLU	(None, 512)	LeakyReLU	(None, 1024)
BatchNormalization	(None, 512)	Dropout	(None, 1024)
Dense_3 (Dense)	(None, 1024)	Dense_3 (Dense)	(None, 512)
LeakyReLU	(None, 1024)	LeakyReLU	(None, 512)
BatchNormalization	(None, 1024)	Dropout	(None, 512)
Dense_4 (Dense)	(None, 53×53)	Dense_4 (Dense)	(None, 256)
LeakyReLU	(None, 53×53)	LeakyReLU	(None, 256)
		Dropout	(None, 256)
		Dense_5 (Dense)	(None, 1)

## Data Availability

The following information was supplied regarding data availability: The IoT Sentinel dataset is available at GitHub https://github.com/andypitcher/IoT_Sentinel/blob/master/iot_fingerprint.py (accessed on 21 May 2026). The UNSW dataset is available at UNSW IoT Analytics: https://iotanalytics.unsw.edu.au/iottraces.html (accessed on 3 May 2026).

## References

[1] Statista Research Department (2019). Internet of Things-Number of Connected Devices Worldwide 2015–2025.

[2] Fan L., Zhang S., Wu Y., Wang Z., Duan C., Li J., Yang J. (2020). An iot device identification method based on semi-supervised learning. 2020 16th International Conference on Network and Service Management (CNSM).

[3] Nguyen-An H., Silverston T., Yamazaki T., Miyoshi T. (2020). Entropy-based iot devices identification. 2020 21st Asia-Pacific Network Operations and Management Symposium (APNOMS).

[4] Sun Y., Fu S., Zhang S., Zhu H., Li Y. (2020). Accurate iot device identification from merely packet length. 2020 16th International Conference on Mobility, Sensing and Networking (MSN).

[5] Perdisci R., Papastergiou T., Alrawi O., Antonakakis M. (2020). Iotfinder: Efficient large-scale identification of iot devices via passive dns traffic analysis. 2020 IEEE European Symposium on Security and Privacy (EuroS&P).

[6] Charyyev B., Gunes M.H. (2020). Iot traffic flow identification using locality sensitive hashes. 2020 IEEE International Conference on Communications (ICC).

[7] Yu C., Li Y., Wang H. (2023). Iot device traffic classification algorithm based on sliding time window. Comput. Eng..

[8] Msadek N., Soua R., Engel T. (2019). Iot device fingerprinting: Machine learning based encrypted traffic analysis. 2019 IEEE Wireless Communications and Networking Conference (WCNC).

[9] Li R., Duan P., Shen M., Zhu L. (2022). Iot device traffic classification algorithm based on random forest. J. Beijing Univ. Aeronaut. Astronaut..

[10] Kostas K., Just M., Lones M.A. (2022). Iotdevid: A behavior-based device identification method for the iot. IEEE Internet Things J..

[11] Cheng S., Wang S., Zhang Y., Zhang Z., Wang Z., Wang B. (2023). Identification of industrial control networked devices based on traffic classification. Comput. Eng. Des..

[12] Miettinen M., Marchal S., Hafeez I., Asokan N., Sadeghi A.-R., Tarkoma S. (2017). Iot sentinel: Automated device-type identification for security enforcement in iot. 2017 IEEE 37th International Conference on Distributed Computing Systems (ICDCS).

[13] Li W., Li Q., Liu R. (2021). Iot devices identification based on machine learning. 2021 IEEE 21st International Conference on Communication Technology (ICCT).

[14] Ammar N., Noirie L., Tixeuil S. (2020). Autonomous identification of iot device types based on a supervised classification. 2020 IEEE International Conference on Communications (ICC).

[15] Bezawada B., Bachani M., Peterson J., Shirazi H., Ray I., Ray I. Behavioral fingerprinting of iot devices. Proceedings of the 2018 Workshop on Attacks and Solutions in Hardware Security.

[16] Ali Z., Hussain F., Ghazanfar S., Husnain M., Zahid S., Shah G.A. (2021). A generic machine learning approach for iot device identification. 2021 International Conference on Cyber Warfare and Security (ICCWS).

[17] Song Y., Qi X., Huang Q., Hu A., Yang J. (2020). Iot device identification algorithm based on two-stage multiclass classification. J. Tsinghua Univ. (Sci. Technol.).

[18] Aksoy A., Gunes M.H. (2019). Automated iot device identification using network traffic. 2019 IEEE International Conference on Communications (ICC).

[19] Yin F., Yang L., Ma J., Zhou Y., Wang Y., Dai J. (2021). Identifying iot devices based on spatial and temporal features from network traffic. Secur. Commun. Netw..

[20] Yin F., Yang L., Wang Y., Dai J. (2021). Iot etei: End-to-end iot device identification method. 2021 IEEE Conference on Dependable and Secure Computing (DSC).

[21] Chen Q., Du Y., Han Y. (2021). Iot device recognition model based on depthwise separable convolution. Netinfo Secur..

[22] Hao Q., Rong Z., Xie L., Hang F. (2023). Online iot device recognition method based on bi-lstm. J. Xi’An Univ. Sci. Technol..

[23] Zhao R., Huang Y., Deng X., Shi Y., Li J., Huang Z., Wang Y., Xue Z. (2023). A novel traffic classifier with attention mechanism for industrial internet of things. IEEE Trans. Ind. Inform..

[24] Wu B., Gysel P., Divakaran D.M., Gurusamy M. (2024). Zest: Attention-based zero-shot learning for unseen iot device classification. 2024 IEEE Network Operations and Management Symposium (NOMS).

[25] Qing G., Wang H., Guo L., Yang J. (2020). Device type identification via network traffic and lightweight convolutional neural network for internet of things. IEEE Access.

[26] Sivanathan A., Sherratt D., Gharakheili H.H., Radford A., Wijenayake C., Vishwanath A., Sivaraman V. (2017). Characterizing and classifying iot traffic in smart cities and campuses. 2017 IEEE Conference on Computer Communications Workshops (INFOCOM WKSHPS).

[27] Rabbani M., Gui J., Nejati F., Zhou Z., Kaniyamattam A., Mirani M., Piya G., Opushnyev I., Lu R., Ghorbani A.A. (2025). Device identification and anomaly detection in iot environments. IEEE Internet Things J..

[28] Cimino G., Deufemia V. (2025). Sigfrid: Unsupervised, platform-agnostic interference detection in iot automation rules. ACM Trans. Internet Things.

